# PlotXpress, a webtool for normalization and visualization of reporter expression data

**DOI:** 10.12688/f1000research.73641.1

**Published:** 2021-11-08

**Authors:** Elias Brandorff, Marc Galland, Joachim Goedhart

**Affiliations:** 1 University of Amsterdam, Amsterdam, The Netherlands

**Keywords:** Reporter assays, gene expression, luciferase, data visualization, webtool

## Abstract

In molecular cell biology, reporter assays are frequently used to investigate gene expression levels. Reporter assays employ a gene that encodes a light-emitting protein, of which the luminescence is quantified as a proxy of gene expression. Commercial parties provide reporter assay kits that include protocols and specialized detection machinery. However, downstream analysis of the output data and their presentation are not standardized. We have developed plotXpress to fill this gap, providing a free, open-source platform for the semi-automated analysis and standardized visualisation of experimental gene reporter data. Users can upload raw luminescence data acquired from a reporter gene assay with an internal control. In plotXpress, the data is corrected for sample variation with the internal control and the average for each condition is calculated. When a reference condition is selected the fold change is calculated for all other conditions, based on the selected reference. The results are shown as dot plots with a statistical summary, which can be adjusted to create publication-grade plots without requiring coding skills. Altogether, plotXpress is an open-source, low-threshold, web-based tool, that promotes a standardized and reproducible analysis while providing an appealing visualization of reporter data. The webtool can be accessed at: https://huygens.science.uva.nl/PlotXpress/

## Introduction

Reporter gene assays are popular tools to investigate gene expression dynamics in cell biology ( Barriscale *et al.*, 2014; Liu *et al.*, 2009; Schenborn and Groskreutz, 1999). In most systems, the reporter is a gene coding for a protein that emits light when it binds a substrate. An example is the firefly ( *Photinus pyralis*) luciferase that emits light when it binds the substrate luciferin ( Himes and Shannon, 2000). The luminescent light can be detected with dedicated equipment and reflects the expression of the reporter gene.

The expression level of the reporter gene is used as a proxy of the capacity of a DNA sequence to regulate gene expression under various experimental conditions. To this end, the DNA sequence of interest is cloned upstream of the reporter gene in a vector which is subsequently transfected into cells. Reporter gene activity can be compared between variations of a regulatory DNA sequence, for example by removing a transcription factor binding site or by modulating tandem repeat expansions in a promoter sequence ( Rodriguez *et al.*, 2020; Moparthi and Koch, 2020). Another option is to measure the effects on reporter activity of treatments such as pharmaceutical compounds or overexpression of transcription factors that interact with the sequence of interest ( Asamitsu *et al.*, 2021). In addition, reporter assays have been used to study the relation between microRNAs and cancer progression ( Wang *et al.*, 2021; Chengling *et al.*, 2021). In the so-called “Dual-Luciferase ^Ⓡ^ Reporter Assay System” ( Promega), an internal control is used to correct for variations in cell density and transfection efficiency. This internal control is co-transfected and consists of a vector with a constitutively active promoter that drives expression of another luciferase. This second luciferase emits a different color of light and is, in our example, derived from *Renilla reniformis* (sea pansy). Transfection of the internal control is kept the same in each well and reflects transfection efficiency and cellular protein production.

A given sequence of interest is cloned into a luciferase expressing plasmid in order to investigate its activity as a gene promoter. The transfection of an empty vector control, without this sequence, is an important reference condition and required to correct for unintended effects of the plasmid alone. For example, in some vectors, luciferase is expressed under control of an SV40 promoter element. A transcription factor binding site (TFBS), cloned upstream of this promoter will influence its activity and the expression of luciferase. The measured effect is a composite of promoter activity *plus* the TFBS. The empty vector control allows normalization for baseline promoter activity and isolation of the influence of the TFBS alone. Here, we will call the empty vector condition the *reference* condition.

**Figure 1.**
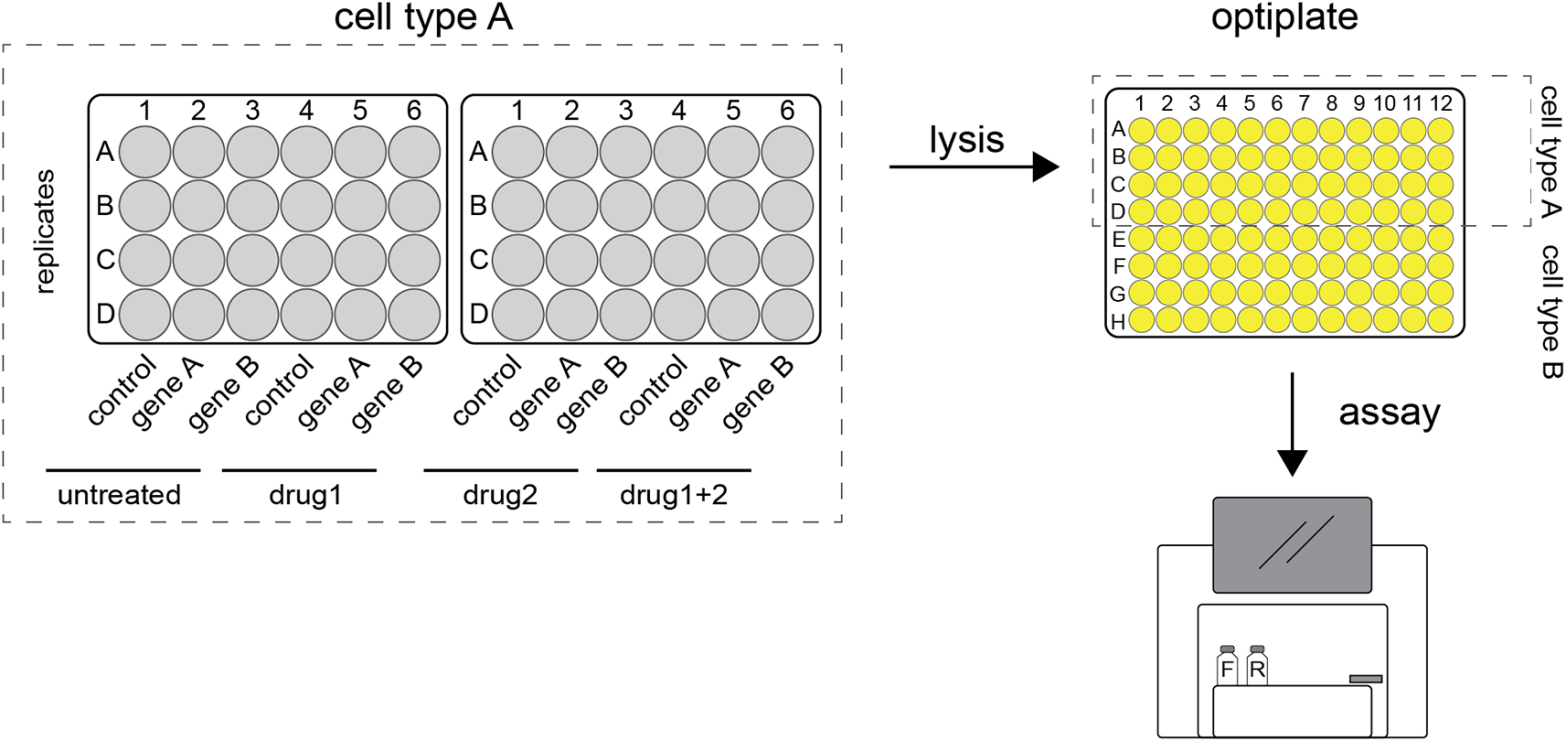
Graphic representation of a typical reporter assay. Firefly and renilla expressing plasmids are transfected into cells grown in 24-well plates. After incubation the cells are harvested and lysates pipetted into 96-wells optiplates. These are loaded into a plate reader where the luciferase substrates are added. Once the reactions are initiated, luminescence is detected, and readings are stored digitally.

In reporter assays with multiple reporter constructs, cell types, treatments and reference conditions, experiments may increase rapidly in size and complexity, leading to challenging downstream analyses *.* There is currently neither a standardized method nor a computational tool for the processing of reporter assay data and its visualization. PlotXpress was designed to simplify the analysis process of complex reporter assays ( Figure 1) by providing an online tool with a standard for data processing and visualization. PlotXpress was built following the philosophy of transparent and state-of-the-art data visualization implemented in the data visualization app PlotsOfData ( Postma and Goedhart, 2019). In addition to a streamlined analysis, plotXpress enables transparent communication of the data. Instead of only providing averaged data and error bars to summarize gene reporter data in bar graphs, plotXpress produces dot plots maintaining individual data points. Data in both wide and tidy format ( Wickham, 2014) can be provided. As coding skills are not required, plotXpress is a readily available low entry-level application that democratizes the processing and visualization of dual reporter expression data.

## Methods

### Implementation

The plotXpress code is written in the R programming language using the following packages: ggplot2 ( Wickham, 2016), tidyr, magrittr, readr, stringr, dplyr and readxl which are all part of the tidyverse suite of packages version 1.3.1 ( Wickham *et al.*, 2019), and Shiny and DT. This manuscript documents version 1.0.0 of the webtool which is archived (together with the example data) at Zenodo ( Goedhart and Galland, 2021).

Background information, updates of the code and version releases will be published on GitHub: https://github.com/ScienceParkStudyGroup/PlotXpress. GitHub is the preferred channel for communication regarding issues and feature requests.

An example dataset with measurements and a dataset with conditions is automatically loaded when the plotXpress app launches. These files, ‘DualLuc_example_data.xlsx’ and ‘Tidy_design.csv’, are also available online ( Goedhart and Galland, 2021).

*Data upload*

Users can upload their data in two different ways. The first option is for data acquired with the Promega GloMax plate reader. The alternative is a general-purpose option that accepts data in a tidy format.

*GloMax data upload*

**Figure 2.**
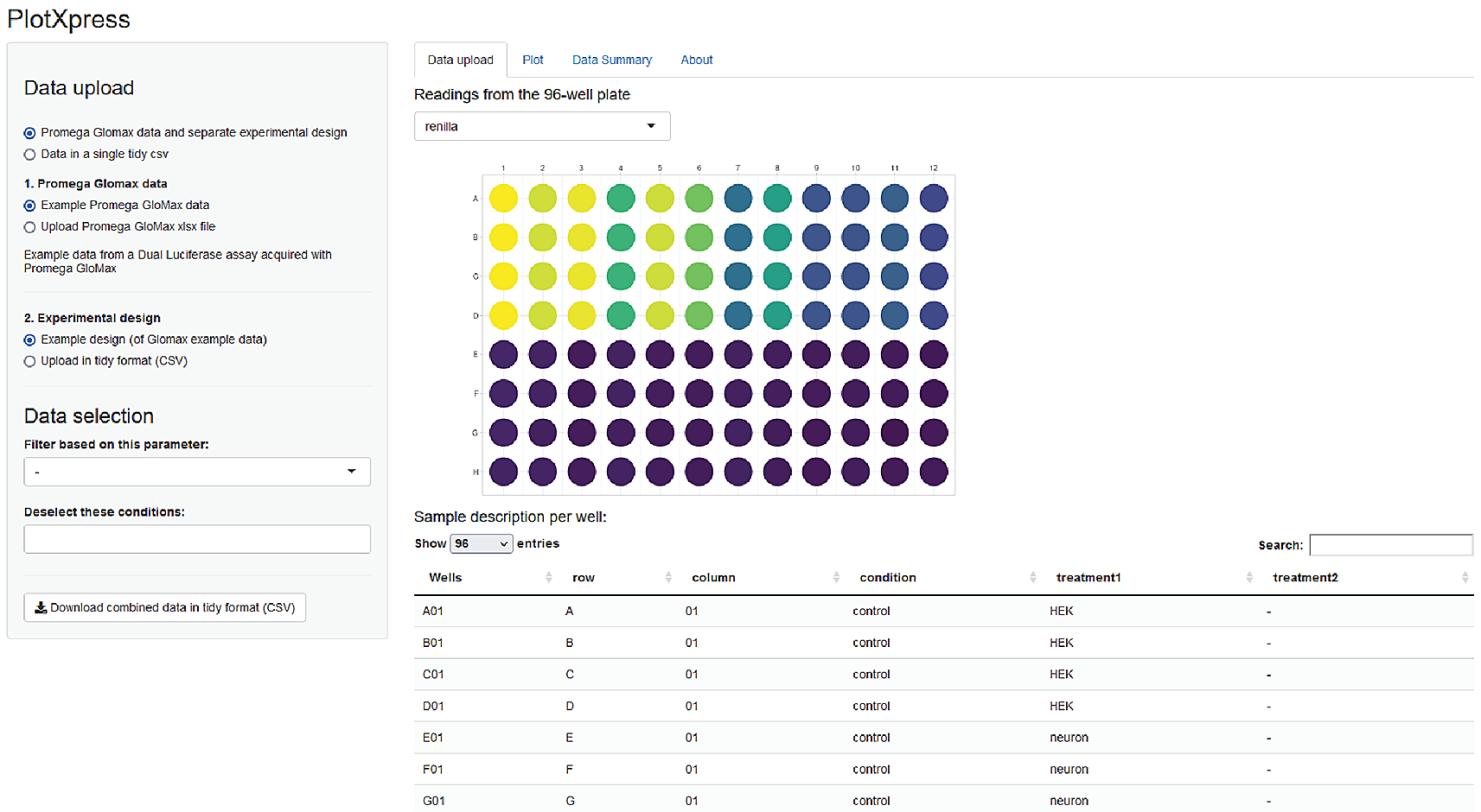
PlotXpress data upload. Screenshot of PlotXpress app showing a 96-well format with signal intensities represented by false colour.

The output of the GloMax plate reader (Promega) is a spreadsheet XLSX format with two tables containing firefly and renilla luminescence signals stored in a 96-well lay-out. PlotXpress reads the cells with the firefly and renilla readings and provides a graphic overview of the experiment by showing a 96-well plate where signal intensity is colour coded ( Figure 2).

**Figure 3.**
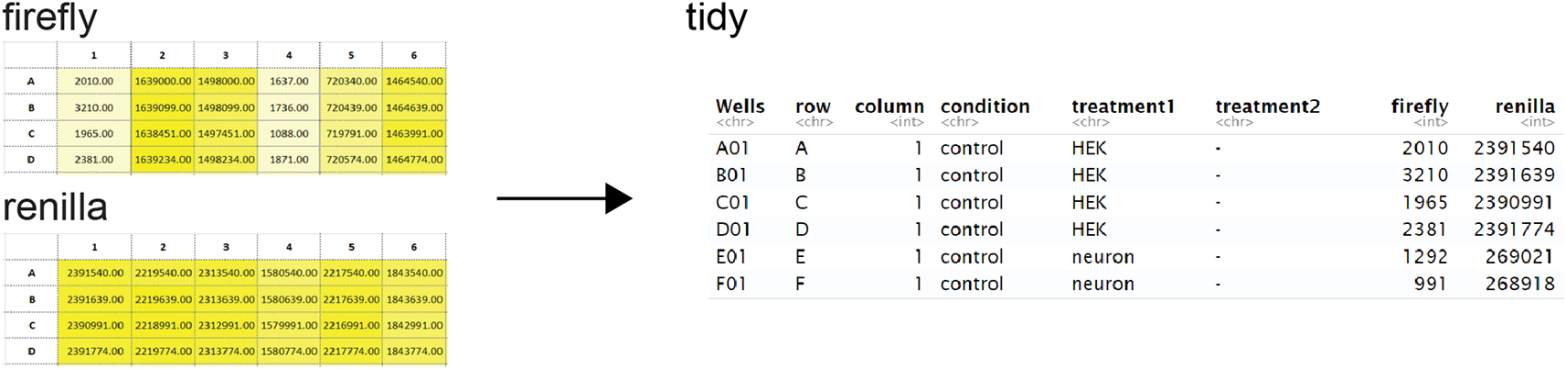
GloMax data conversion to tidy format. PlotXpress allows uploading of GloMax spreadsheet input. An additional experimental design file in tidy format should be provided to connect the reads with the conditions. After supplying the design file, the GloMax data is converted to a table in tidy format, where each row contains a single observation and the experimental conditions. The table can be downloaded and used as input for plotXpress.

The firefly and renilla reads from the GloMax data are converted into a tidy format and merged with experimental conditions that are taken from the uploaded design ( Figure 3). The resulting tidy dataframe is used for data processing.

A separate table in CSV format with the experimental conditions per well is required for the data processing. A template is available for download within the app or online ( Goedhart and Galland, 2021).

*Tidy data upload*

Instead of uploading a GloMax spreadsheet in 96-well format and an additional design file, users can choose to upload a single tidy dataset (.CSV) containing both experimental conditions and firefly and renilla signals. An example file is available online ( Goedhart and Galland, 2021).

All the relevant data from one experiment (design and measurements) should be present in one tidy table. Users are free in the size of the experiment: any number of cell types, sequences of interest, or treatments may be added. The minimal information that is required is a column with wells (in the format A01, B01, ..), a column with intensity data and a column with the conditions. An additional column with reference data is optional and is used to normalize the data if supplied.

*Data processing*

In dual luciferase assays, each replicate consists of a firefly luciferase and renilla luciferase intensity reading. To normalize the reporter gene expression to the internal control, plotXpress calculates the ratio of firefly signal to renilla signal *,* resulting in the firefly/renilla ratio. An average firefly/renilla ratio is then calculated for each group of readings that share identical names in the “condition” column. Finally, the fold change is calculated by dividing the firefly/renilla ratio by the firefly/renilla ration of a selected reference condition:

$$\text{Fold Change}=\frac{\left(\text{firefly}/\text{renilla}\right)}{{\left(\text{firefly}/\text{renilla}\right)}_{\text{reference}}}$$ (1)

### Operation

The plotXpress web tool can be accessed at https://huygens.science.uva.nl/PlotXpress/ or https://goedhart.shinyapps.io/PlotXpress. The online web app runs on any computer platform with a browser and an internet connection. Instructions to run the app locally in R/Rstudio can be found in the README file on the Github page. Upon launching the app, the example data is loaded and the users can choose to visualize the example data by selecting the ‘Plot’ tab or to upload their own data ( Figure 2).

The data upload accepts both GloMax xlsx files, as well as tidy data in CSV format. After data upload, PlotXpress offers flexibility in the selection of conditions to filter the data before plotting. Individual wells can be removed from the analysis or specific conditions can be filtered.

The combined data from an experiment can be downloaded as a CSV file in tidy format. This data format can be used for further processing, such as statistical testing and plotting. The tidy format is well handled by statistical software (R) and other web tools that we have developed (available online). For instance, when the data consists of a mix of technical and biological replicates, this can be visualized as a superplot ( Lord *et al.*, 2020) by uploading the data in the SuperPlotsOfData app ( Goedhart, 2020).

**Figure 4.**
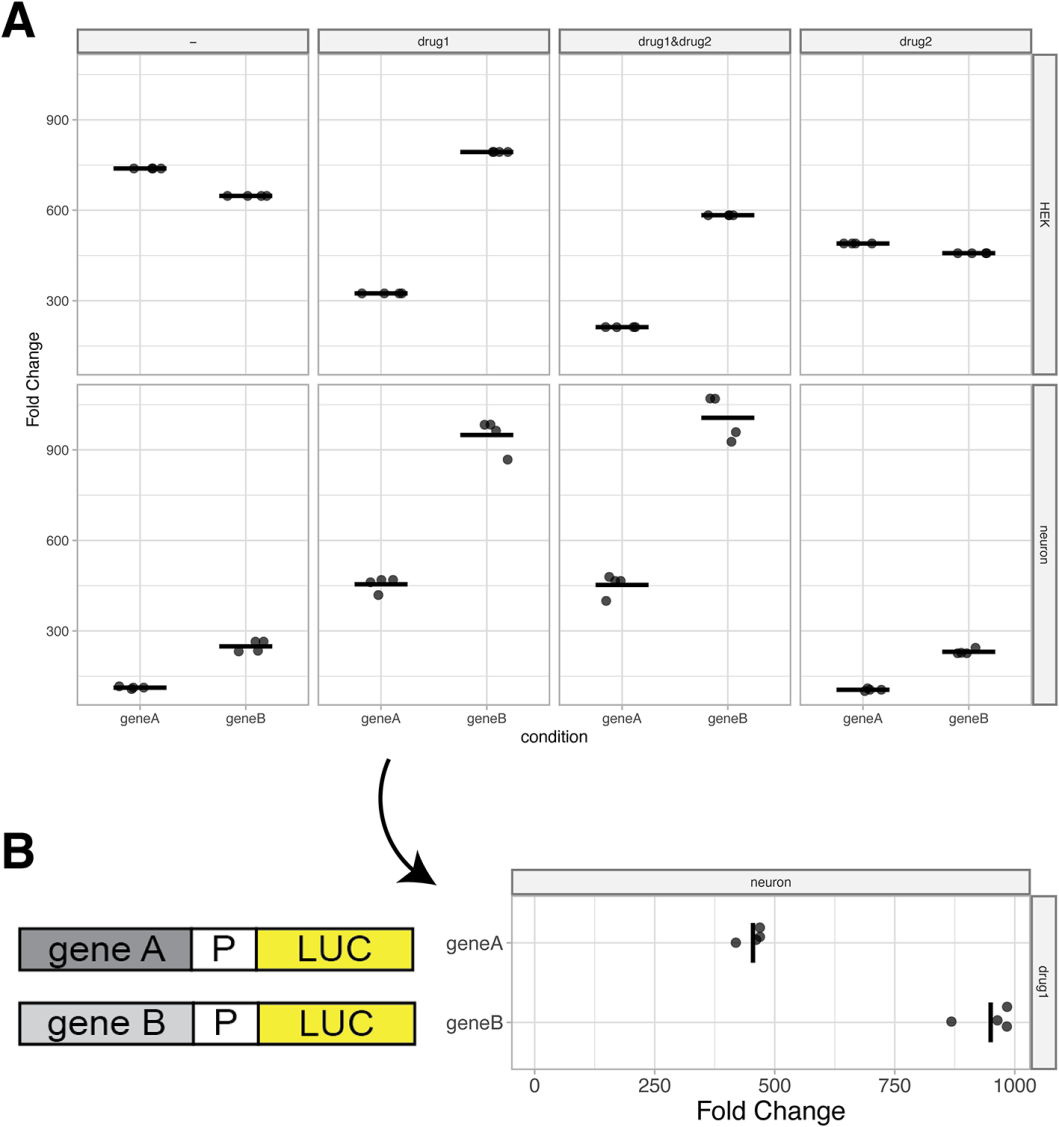
Plots based on example data. (A) Standard output plot produced by PlotXpress after selecting a reference condition. The plots show the fold change in expression induced by gene A and gene B relative to a reference, for each of 8 different conditions. Individual datapoints are shown and the median of each condition is indicated with a horizontal line. (B) A 90° rotated panel for one of the conditions (drug1 in neurons) shown in panel A. Rotation of the panel improves readability of the fold change and allows for the display of a cartoon of the reporter gene next to it.

To visualize the data, users can select the ‘Plot’ tab. First, a reference condition should be selected. This is generally a control condition, e.g. an empty vector in a reporter assay. Without selecting a reference, the firefly/renilla ratio is plotted on the y-axis. If a reference condition is selected, normalized firefly/renilla values from the other conditions are represented as fold-change relative to the reference ( Figure 4A). As an optional feature, the reference can be shown or hidden, depending on user preference.

Several options are available to optimize the data visualization. The size and transparency of the datapoints can be adjusted, gridlines can be removed, summary statistics can be selected, and the font size and plot size can be changed. The plots can be downloaded from PlotXpress in PNG or PDF format, the latter being ideal for downstream processing with vector-based graphic software.

PlotXpress also produces a table with summary statistics that can be found under the ‘Data Summary’ tab. It can be downloaded in multiple formats such as CSV or PDF.

## Use cases

The plotXpress app automatically loads an experimental dataset from a GloMax dual-luciferase reporter assay. This set contains 96 measurements acquired from different conditions. The conditions differ in the vector that is transfected (control, geneA or geneB), in the drug treatment (no drug, drug1, drug2 or drug1&drug2) and the cell type (Hek or neuron). Three measurements were done for each unique condition. The ‘control’ is set as a reference condition and the ‘geneA’ and ‘geneB’ condition are compared to the reference and their levels are expressed as ‘Fold Change’ ( Figure 4A). The resulting data visualization can be used to compare the effect of geneA and geneB relative to the empty vector control and under different conditions. The display of the actual datapoints contributes to the transparency in the communication of the results.

When presenting reporter expression data, it can be useful to display a rotated plot. Diagrams of reporter constructs can be included to visually support the experimental set up and show the corresponding measurements in line ( Figure 4B).

**Figure 5.**
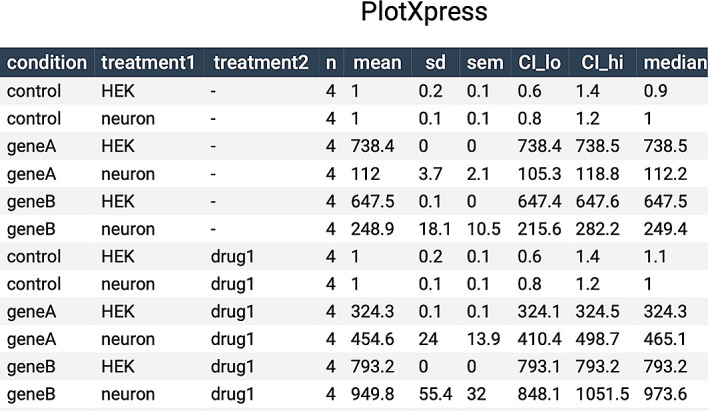
Data summary table. Table containing summary statistics of the data shown in Figure 4A.

A summary of the data is available under the ‘Data Summary’ tab ( Figure 5) and this table can be included as supplemental data in publications.

## Conclusion

Gene reporter assays are an invaluable tool for molecular biology, enabling the study of regulatory DNA sequences, transcription factors, pharmaceutical compounds, oligonucleotides and other factors that interact with DNA sequences. With each treatment or cellular context, the assay increases in complexity. PlotXpress simplifies the data analysis by bridging the gap between wet-lab standards (96-well format) and dry-lab conventions (tidy data format) that are typically used in downstream analysis of experimental biological data.

The current version of plotXpress was written to analyze expression data in tidy format as well as for GloMax output data. The import of data produced by plate readers from other manufacturers is not supported at this moment, but we welcome suggestions and example data to implement import functions for other data formats. Since plotXpress is open-source, users can modify the source code by making a fork to the GitHub repository and create a pull request to have their changes reviewed and possibly integrated. Although plotXpress was developed for the analysis and visualization of dual-luciferase experiments, it can be used for other types of data that require normalization or comparison with a reference condition, such as quantitative polymerase chain reaction data.

The open-source web tool is freely accessible online, democratizing the state-of-the art data visualization of reporter assay data. Moreover, the standardized data processing will contribute to transparent and reproducible science.

## Data availability

### Underlying data

All data underlying the results are available as part of the article and no additional source data are required.

### Extended data

Zenodo: ScienceParkStudyGroup/PlotXpress: v1.0.1 https://doi.org/10.5281/zenodo.5554885 ( Goedhart and Galland, 2021).

This project contains the following files:

- DualLuc_example_data.xlsx. Example data file
- Tidy_design.csv. Example conditions file

Data are available under the terms of the Creative Commons Attribution 4.0 International license (CC-BY 4.0).

## Software availability

Software available from: https://huygens.science.uva.nl/PlotXpress/

Also available from: https://goedhart.shinyapps.io/PlotXpress/

Source code available from: https://github.com/ScienceParkStudyGroup/PlotXpress/tree/v1.0.0

Archived source code at time of publication: https://doi.org/10.5281/zenodo.5554885 ( Goedhart and Galland, 2021)

License: Apache License v2.0

## Author contributions

E.B. initiated the project, contributed experimental data, wrote code and wrote the manuscript. M.G. supervised the project, wrote code, reviewed code and edited the manuscript. J.G. wrote code and edited the manuscript.
