# Supplementary material for: PlotXpress, a webtool for normalization and visualization of reporter expression data
Source: F1000Res. 2021 Nov 8;10:1125. [Version 1] doi: 10.12688/f1000research.73641.1 (PMC8630550; doi:10.12688/f1000research.73641.1)
Supplement: Peer review history

# Peer review: Reviewer response for version 1

**DOI:** 10.5256/f1000research.77305.r99321

**Published:** 2021-12-14

**Authors:** Vincent J. Lynch

**Affiliations:** 1 Department of Biological Sciences, University at Buffalo, Buffalo, NY, USA

PlotXpress is well explained and documented, and has a very user friendly web application. I see no need for revisions for other changes to either the method or the manuscript describing the results. This will be extremely useful for those using Luciferase assays and other methods that use a plate.

Are the conclusions about the tool and its performance adequately supported by the findings presented in the article?

Yes

Is the rationale for developing the new software tool clearly explained?

Yes

Is the description of the software tool technically sound?

Yes

Are sufficient details of the code, methods and analysis (if applicable) provided to allow replication of the software development and its use by others?

Yes

Is sufficient information provided to allow interpretation of the expected output datasets and any results generated using the tool?

Yes

Reviewer Expertise:

Evolutionary biology.

I confirm that I have read this submission and believe that I have an appropriate level of expertise to confirm that it is of an acceptable scientific standard.

# Peer review: Reviewer response for version 1

**DOI:** 10.5256/f1000research.77305.r99317

**Published:** 2021-11-29

**Authors:** Guillaume Jacquemet

**Affiliations:** 1 Turku Bioscience Centre, University of Turku, Turku, Finland; 2 Abo Akademi University, Turku, Finland

In this article, Brandorff *et al.* describe a tool to visualize and display reporter expression data. PlotXpress is very easy to use and appears to be well documented. I particularly like the visualization of the dataset using the 96 well-plate layout, which is very intuitive and will prove very useful when analyzing HTS datasets. Below I outline several points the authors could consider when developing their tool further in the hope they find them helpful.

 The authors describe PlotXpress as a tool to visualize luciferase-based reporter expression data. Why being so restrictive? I can easily imagine that this tool can also be used to visualize any time of dataset acquired in a 96 well-format (drug screening, Elisa binding assays, etc.). This could be easily reflected in the title and throughout the manuscript.

 While the tool is already very powerful, I think the most significant factor that will limit its adoption is the data format required to import the dataset. Tidy CSV formats are painful to create in the best of time. If possible to implement, it would be great to be able to import multiple CSV files (where each file is an individual repeat). It would be helpful to provide example input files on the PlotXpress website directly.

 Regarding the data visualization, it may be useful to allow the users to know control what is displayed in the color-coded 96-well plate. I am assuming that the average signal is currently displayed in the plot by default. But showing the standard variation (or other parameters) could be equally valuable to identify conditions with high variability. 

 Regarding the normalization options, It could be helpful to provide the possibility to normalize the data to the overall detected signal in the plate for each repeat. This can sometimes be more beneficial than normalization to CTRL wells alone. It would also be beneficial for the quantifications to compute the Z-score, which is often used for HTS analyses.

Are the conclusions about the tool and its performance adequately supported by the findings presented in the article?

Yes

Is the rationale for developing the new software tool clearly explained?

Yes

Is the description of the software tool technically sound?

Yes

Are sufficient details of the code, methods and analysis (if applicable) provided to allow replication of the software development and its use by others?

Yes

Is sufficient information provided to allow interpretation of the expected output datasets and any results generated using the tool?

Yes

Reviewer Expertise:

Cell biology

I confirm that I have read this submission and believe that I have an appropriate level of expertise to confirm that it is of an acceptable scientific standard.

# Peer review: Reviewer response for version 1

**DOI:** 10.5256/f1000research.77305.r99324

**Published:** 2021-11-24

**Authors:** Lorena Pantano Rubino

**Affiliations:** 1 NextRNA Therapeutics, Boston, MA, USA

PlotXpress is well explained and documented. It has a clear rational about the need and the solution it brings. Authors have developed the application following best practices. It has a good explanation on installation and usage. I have only a few minor comments that should be easily addressed:

1. Adding the possibility to make the plots interactive could be helpful to evaluate replicates. For instance, if there is a bad replicate, hovering over the dots should make easy to know which one is the issue or needs further discussion.
2. Adding information on the README on how to contribute, authors can look at bigger projects to mimic the best practices for this.
3. Fix the install line code in the README to render in the proper way.
4. Not sure why the README contains the gadgets from shiny package, I don't think they are needed here since they point to the test for the shiny app, not this package.
5. Is the rsconnect folder necessary in the repository?

Are the conclusions about the tool and its performance adequately supported by the findings presented in the article?

Yes

Is the rationale for developing the new software tool clearly explained?

Yes

Is the description of the software tool technically sound?

Yes

Are sufficient details of the code, methods and analysis (if applicable) provided to allow replication of the software development and its use by others?

Yes

Is sufficient information provided to allow interpretation of the expected output datasets and any results generated using the tool?

Yes

Reviewer Expertise:

Bioinformatics, R, shiny, visualization, gene regulation,

I confirm that I have read this submission and believe that I have an appropriate level of expertise to confirm that it is of an acceptable scientific standard.
